# Aberrant Anatomical Variation of Maxillary Sinus Mimicking Periapical Cyst: A Report of Two Cases and Role of CBCT in Diagnosis

**DOI:** 10.1155/2013/757645

**Published:** 2013-04-24

**Authors:** Ahmet Ercan Sekerci, Yildiray Sisman, Meryem Etoz, Duygu Goller Bulut

**Affiliations:** Department of Oral and Maxillofacial Radiology, Faculty of Dentistry, Erciyes University, 38039 Kayseri, Turkey

## Abstract

Most periapical lesions are associated with microorganisms from infected root canal systems. Maxillary sinus can pose a diagnostic dilemma radiographically because of its anatomical variation which can mimic a periapical pathosis. The aim of this study was to describe two cases of aberrant anatomical variation of the maxillary sinus that presented radiographic similarities to a periapical cyst in order to call the attention of clinicians to the fact that several different diseases are able to mimic endodontic periapical lesions. An accurate assessment of this morphology was made with the help of cone-beam computed tomography (CBCT).

## 1. Introduction

 Several cases of benign and malignant lesions mimicking radiographically periapical inflammatory lesions, such as carcinoma [1], odontogenic cyst [2], periapical cemental dysplasia [3], benign tumors, and locally aggressive or malignant neoplasias [4], have been described in the literature. Film-processing errors have also been reported to mimic the appearance of periapical infection and cause diagnostic confusion [5]. Plain radiographs play an important role in the management of dentomaxillofacial lesions especially for detection, treatment, and followup of bone lesion. However, routine radiographic procedures do not demonstrate reliably the presence of every lesion. Furthermore, advanced imaging techniques can influence the diagnosis [6].

In the present report, two unique cases of anatomical variation of the maxillary sinus manifesting as a well-defined periapical radiolucency in relation to the roots of the upper left first molar and right second premolar, which were initially misdiagnosed as a periapical cyst, are described. This variation may have been interpreted as a periapical inflammatory lesion. The value of cone-beam computed tomography (CBCT) as a prognostic determinant was highlighted.

## 2. Case Report

### 2.1. Patient I

**Figure 1:**
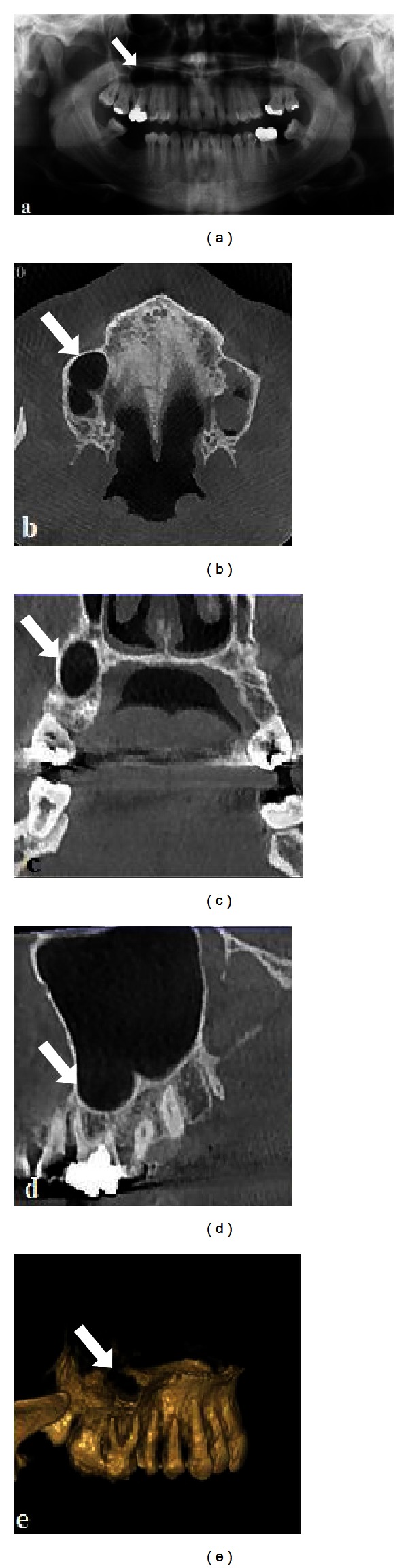
Panoramic radiograph showing well-defined periapical radiolucency in relation to maxillary first molar, extending to involve the root of canine and suggestive of a periapical cyst (a). Axial (b), coronal (c), sagittal, and three dimensional view of the aberrant anatomical variation of maxillary sinus.

A 25-year-old man was referred by his general dental practitioner to the Department of Oral and Maxillofacial Radiology regarding a periapical lesion related to the apex of the upper right molar teeth and prosthetic management. Intraoral examination revealed that there were a few teeth with caries in different regions. Panoramic radiography revealed a well-defined radiolucency involving the root apices of the upper right maxillary first molar (Figure 1(a)). The borders exhibited dense radiopaque features. Provisional diagnosis of the radicular cyst was established, and root canal treatment was carried out. Endodontic retreatment was initiated by his dentist, but after a followup of several months, no changes were observed in the ovoid radiolucency, and the patient was referred for consultation to our department. The patient's medical history was unremarkable, included no trauma of the jaws, and he had not undergone any jaw surgery. He was not aware of the radiolucent area before his routine dental examination and had no sensory or motor deficiency. The tooth was asymptomatic and it also presented with a poor root canal filling. The overlying mucosa of the lesion was quite normal, and there was no sign of infection or fistula. The overall appearance of the lesion seemed to be that of a radicular cyst of the right maxillary first molar. On performance of vitality tests, all the teeth except the first molar- in the mentioned area appeared to be vital.

To reveal the exact location definition of the pathologic features, he was referred for a CBCT (NewTom 5G, QR, Verona, Italy) scan. After this, a CBCT was taken which revealed the maxillary sinus with its border extending but not involving the roots of the mentioned teeth, which is one of the anatomical variations of maxillary sinus (Figure 1(b)–1(e)). The dimensions of the defect were 13.2 × 14.2 × 9.3 mm depth (mesiodistal length, inferosuperior height, and buccolingual depth).

### 2.2. Patient II

**Figure 2:**
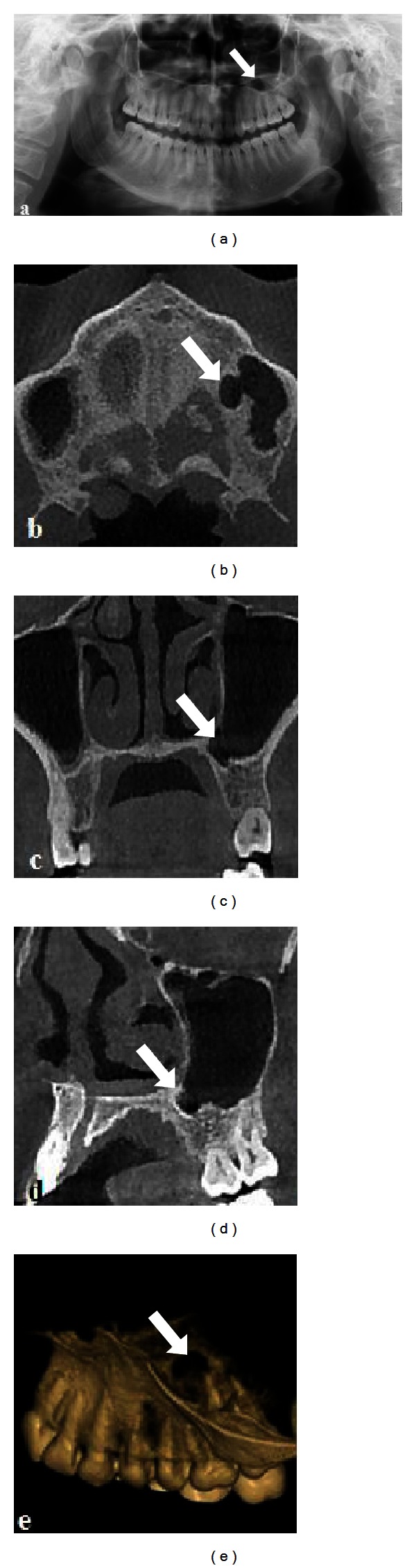
Panoramic radiograph showing well-defined periapical radiolucency in relation to apices of the maxillary left second premolar and first molar (a). Axial (b), coronal (c), sagittal, and three dimensional view of the anatomical variation of maxillary sinus.

A 29-year-old otherwise healthy woman was referred to our clinic for routine dental examination and periodontal management. Panoramic radiography showed a unilocular, oval, and radiolucent lesion with well-defined sclerotic borders situated at the apices of the second left premolar and first molar teeth in the maxilla (Figure 2(a)). The radiolucency was located superior to, but not continuous with, the periodontal area of the tooth. The patient's history included no trauma of the jaws, and she had not undergone any jaw surgery. The patient was not aware of the radiolucent area before her routine dental examination and had no sensory or motor deficiency, and there was no pain. The overlying mucosa of the lesion was quite normal, and there was no sign of infection or fistula. The overall appearance of the lesion seemed to be that of a radicular cyst of the right maxillary first molar, On performance of vitality tests, all the teeth in the mentioned area appeared to be vital. 

To reveal the exact location definition of the pathologic features, she was referred for cone-beam computed tomography (CBCT: Newtom 5G, QR, Verona, Italy) scan. The dimensions of the defect were 7.3 × 6.7 × 4.7 mm depth (mesiodistal length, inferosuperior height, and buccolingual depth).

Then by combining the results of the patients from clinical examination, radiographic examinations, results from vitality tests, and CBCT examination of the patients, the final diagnosis was made as one of the anatomical variations of the maxillary sinus (Figures 1 and 2).

## 3. Discussion

The present report deals with one such diagnostic problem, where a maxillary sinus was interpreted in a panoramic radiograph as a periapical lesion. The maxillary sinus is a pyramid-shaped cavity with thin walls corresponding to the orbital, alveolar, facial, and infratemporal aspects of the maxilla. The size, shape, and wall thickness of the sinus vary from one to another even on the two sides of an individual skull [7]. It develops by the invagination of mucous membrane from the nasal cavity. The borders of the maxillary sinus appear on periapical radiographs as a thin, delicate, and tenuous radiopaque line (actually a thin layer of cortical bone). In the absence of disease, it appears continuous, but on close examination, it can be seen to have small interruptions in its smoothness or density. These discontinuities are probably illusions caused by the superimposition of small marrow spaces [8]. Superiorly, the roof is formed by the orbital plate and inferiorly it is bordered by the alveolar process of maxilla [5].

The degree of extension of the maxillary sinus into the alveolar process is extremely variable. The floor of the maxillary sinus is seen on dental radiographs at approximately the same level around the age of puberty. In some projections the floor of the sinus will be well above the apices of the posterior teeth. The roots of the molars usually lie in close apposition to the maxillary sinus. Root apices may project anatomically into the floor of the sinus, causing small elevations or prominences. The thin layer of bone covering the root is seen as a fusion of the lamina dura and the floor of the sinus. When the rounded sinus floor dips between the buccal and palatal molar roots and is medial to the premolar roots, the projection of the apices is superior to the floor. This appearance conveys the impression that the roots project into the sinus cavity, which is an illusion [8]. There has been one case reported in which the maxillary sinus extended from the lateral incisors to the third molars [9]. The sinus floor usually has its most inferior point near the first molar region. Several conical elevations projecting into the sinus floor and corresponding to the roots of the first and second molar teeth sometimes perforate [10].

Frequently one or a number of radiopaque lines which are called septa traverse the image of the maxillary sinus. They are thin folds of cortical bone that project a few millimeters away from the floor and wall of the antrum, or they may extend across the sinus. They appear on many periapical intraoral radiographs and commonly on CBCT images. While septa appear to separate the sinuses into distinct compartments, this is rarely the case. Rather, the septa naturally extend only a few millimeters into the central volume of the sinus. Septa deserve attention because they sometimes mimic periapical disease, and the chambers they create in the alveolar recess may complicate the search for a root fragment displaced into the sinus [8].

Maxillary cysts are pathologic cavities with a liquid or semiliquid content delimited wholly or partially by epithelium; the origin of the latter allows one to distinguish cysts as odontogenic or nonodontogenic [11]. Radicular cysts are considered to be lesions of an odontogenic nature which originate from the epithelial residues present in the parodontium space, and their proliferation is activated by an inflammatory- type mechanism [12]. The radicular cyst is the most common cystic lesion, with a frequency of about 50% amongst all cystic lesions of odontogenic nature. This lesion is often diagnosed accidentally while taking normal radiographic tests. A rather convenient indication to address the diagnoses is the vitality test, as a radicular cyst is always associated with a necrotic dental element [13]. From a radiographic point of view, this neoformation appears as a unilocular radio transparency with very clear margins with a roundish shape still in relation with the radicular apex [14].

Basnet et al. [5] suggested that, when periapical radiolucency is seen in theradiograph, it may be a distinct pathologic lesion or it could just be an anatomical variation. The gathering of data by clinical and radiographic examinations helps clinicians to form an operational opinion. They also stated that only endodontic therapy should be initiated if these data support the diagnosis of radiolucent lesion associated with a nonvital tooth. If it does not, then further diagnostic data should be collected from various clinical tests, advance imaging techniques, biopsy, consultation with appropriate specialists, and so forth. In the present study, on performance of vitality tests, all the teeth appeared to be vital. Therefore, CBCT scan was performed for the diagnosis.

Therefore, thorough knowledge about the anatomy of the maxillary sinus and its variations, proper diagnostic aids and good interpretation skills are essential to form an accurate diagnosis.

## 4. Conclusion

The present report highlights the need for careful consideration of the causes of periapical radiolucency before making an actual diagnosis using CBCT and identifies a poorly recognized anatomical variation which may cause diagnostic confusion. In addition, it is necessary for dentists to be reasonably well informed on the radiographic appearances of the normal anatomy, as well as the abnormalities and diseases that occur in the maxilla. 

## References

[1] Cutler R (1990). Neoplasia masquerading as periapical infection. *British Dental Journal*.

[2] Hancock MAF, Brown CE, Hartman KS (1986). Orthokeratinized odontogenic cyst presenting as a periapical lesion. *Journal of Endodontics*.

[3] Smith S, Patel K, Hoskinson AE (1998). Periapical cemental dysplasia: a case of misdiasnosis. *British Dental Journal*.

[4] García CC, Sempere FV, Diago MP, Bowen EM (2007). The post-endodontic periapical lesion: histologic and etiopathogenic aspects. *Medicina Oral, Patología Oral y Cirugía Bucal*.

[5] Basnet P, Kamath MP, Kundabala M, Menda A (2005). Anatomical variation of maxillary sinus mimicking a periapical cyst: a case report. *Kathmandu University Medical Journal*.

[6] Saunders MB, Gulabivala K, Holt R, Kahan RS (2000). Reliability of radiographic observation recordedon a performa using inter and intra observer variation: a preliminary study. *International Endodontic Journal*.

[7] Arman C, Ergür I, Atabey A (2006). The thickness and the lengths of the anterior wall of adult maxilla of the West Anatolian Turkish people. *Surgical and Radiologic Anatomy*.

[8] White SC, Pharoah MJ (2009). *Oral Radiology Principles and Interpretation*.

[9] Srinivasan B (2004). *Textbook of Oral and Maxillofacial Surgery*.

[10] van den Bergh JPA, ten Bruggenkate CM, Disch FJM, Tuinzing DB (2000). Anatomical aspects of sinus floor elevations. *Clinical Oral Implants Research*.

[11] Ciulli E, Rocci M, Bollero R (2009). Maxillary cyst: description of a clinical case. *Oral Implantology*.

[12] Ricucci D, Pascon EA, Pitt Ford TR, Langeland K (2006). Epithelium and bacteria in periapical lesions. *Oral Surgery, Oral Medicine, Oral Pathology, Oral Radiology and Endodontology*.

[13] Murmura G, Traini T, di Iorio D, Varvara G, Orsini G, Caputi S (2004). Residual and inflammatory radicular cysts. Clinical and pathological aspects of 2 cases. *Minerva Stomatologica*.

[14] Düker J (2005). Radiographic diagnostics. Radicular cyst. *Quintessence International*.

